# Distinct Impacts of Pre-Operative Antiviral Treatment on Post-Operative Outcomes of HBV-related Hepatocellular Carcinoma: A Landmark Analysis

**DOI:** 10.7150/jca.47125

**Published:** 2021-01-01

**Authors:** Jun Fu, Zongren Ding, Qinjunjie Chen, Kongying Lin, Hongzhi Liu, Yuzhen Gao, Yongyi Zeng, Haitao Li, Feng Shen, Jingfeng Liu

**Affiliations:** 1 Department of Hepatopancreatobiliary Surgery, Mengchao Hepatobiliary Hospital of Fujian Medical University, Fuzhou, China.; 2 The Big Data Institute of Southeast Hepatobiliary Health Information, Fuzhou, China.; 3 Department of Hepatic Surgery IV, the Eastern Hepatobiliary Surgery Hospital, Naval Medical University, Shanghai, China.; 4 Department of Laboratory Medicine, the Eastern Hepatobiliary Surgery Hospital, Naval Medical University, Shanghai, China.

**Keywords:** Anti-viral treatment, HBV-related HCC, Surgery, Long-term Prognosis

## Abstract

**Background:** The effect of anti-viral treatment (AVT) initiated before surgery (pre-operative AVT) on HBV-related hepatocellular carcinoma (HCC) has been controversial. This study aimed to elucidate the prognostic significance of pre-operative AVT for HCC patients who received hepatectomy.

**Materials and Methods:** A large-scale retrospective study was conducted based on a cohort consisting of 1937 HBV-related HCC patients who underwent R0 liver resection between January 2011 and December 2012. Propensity score matching (PSM) method was adopted to balance covariates and landmark survival analyses were performed to visualize effects in different phases after surgery.

**Results:** After PSM, a total of matched 744 patients (372 in each group) were recruited. The patients in the pre-operative AVT group had lower HBV-DNA loading levels and better recurrence-free survival (RFS) than those in the non-AVT group. The 1, 3, 5-year RFS rates of two groups were 67.3%, 49.0%, and 43.1% vs. 66.7%, 41.1% and 18.5%, respectively (*P<*0.001). Landmark survival analyses demonstrated that pre-operative AVT could improve RFS, and the effect was beginning to show after the first 12 months. There was no significant difference of overall survival (OS) between the two groups (*P=*0.543), and the landmark survival analyses indicated that pre-operative AVT could improve OS and this effect was beginning to show after 36 months. Additionally, multivariate Cox regression analyses revealed that larger tumor (>5cm), esophageal and gastric varices, lymph node metastasis were independent risk factors of RFS, and larger tumor (>5cm) and ascites were independent risk factors of OS.

**Conclusions:** Pre-operative AVT could significantly improve the RFS, and could not improve short-term OS (< 36 months) but could better long-term survival of the patients with HBV-HCC after surgery.

## Introduction

Primary liver cancer (PLC) has become the fifth leading lethal cause and the second leading cause of cancer-related deaths around China by 2017 1, 2. The main histological origin of hepatic malignancy is hepatocellular carcinoma (HCC), which occurs in hepatocytes and accounts for the majority of PLC 3. Despite recent advances in screening and surveillance of the high-risk population, along with improvements of diagnostic measures and perioperative management, deaths by HCC continue to increase, imposing a heavy public health burden 4. The etiological landscape of HCC worldwide has shifted in past decades, in which hepatitis B virus (HBV) and hepatitis C virus (HCV) infection remain the two main risk factors, while the number of alcohol- or nonalcoholic fatty liver disease (NAFLD)-related HCC patients is increasing 5-7.

Chronic hepatitis B (CHB) is the predominant cause of HCC in Asia and sub-Saharan Africa. In China alone, the rate of CHB exceeds 80% in patients with HCC 8. The national HBV vaccination program has been successful in preventing perinatal transmission in China, resulting in an emerging downward trend of CHB. Additionally, implementation of the vaccine has also potentially decreased the incidence of HBV-related HCC. However, the harsh reality is that vaccine failure and insufficient prevention leave HBV-infected persons still at high risk of HCC, which could not be eradicated by antiviral therapies 9. Currently, radical liver resection (LR) has been regarded as the first-line putative curative approach and the backbone of comprehensive management for HCC 10. For patients with HBV-related HCC, several studies have suggested that patients should receive antiviral therapy after surgery regardless of high or low loads of HBV-DNA, and that most patients might follow their personalized antiviral regimen 11-13.

However, for CHB patients who eventually develop HCC, the impact of antiviral treatment (AVT) initiated before surgery on the prognosis is still controversial, as previous studies paid more attention to tumor characteristics and post-operative adjacent treatment and might underestimate the effectiveness of pre-operative AVT 14, 15. In this study, pre-operative AVT refers to the initial AVT received before diagnosis and hepatectomy of HCC. However, studies conducted to approach this issue have often overlooked the necessity of balancing the baseline data between groups 16, 17. In the present study, a large cohort was introduced to elucidate the short- and long-term prognostic significance of pre-operative AVT on patients with HBV-induced HCC who received R0 resection, and a propensity score matching (PSM) method was adopted to balance the differences of regarding covariates.

## Material and Methods

### Study design and patient populations

A large-scale study was conducted based on a cohort of 1937 patients with HBV-related HCC who underwent hepatectomy between January 2011 and December 2012. These data was extracted from the Primary Liver Cancer Big Date (PLCBD) same to the previous study 18. The inclusion criteria of this study were as follows: 1) HCC underwent R0 resection; 2) HBsAg positivity for more than 6 months; 3) Child-Pugh grade A or B7 (score ≤7 [none to mild compromise]) liver function. The exclusion criteria included: 1) patients received other initial anticancer treatment before surgery such as radiofrequency ablation (RFA), transcatheter arterial chemoembolization (TACE) or sorafenib; 2) accepted interferon as anti-hepatitis B virus treatment before operation; 3) presence of macroscopic vascular invasion; 4) history of other malignancies; 5) concurrent hepatitis C virus antibody positivity; 6) perioperative mortality within 30 days of surgery; 7) missing clinicopathological data. All the eligible individuals were divided into AVT and non-AVT group according to whether or not they had received pre-operative AVT.

### Data collection

All clinicopathological data were collected using PLCBD as comprehensively as possible. General history collection included drinking, smoking, diabetes mellitus (DM), and family history of HCC. In addition, routine pre-operative laboratory testing results were collected including quantitative analysis of serum HBV-DNA level, hepatitis B surface antigen (HBsAg), hepatitis Be antigen (HBeAg), aspartate aminotransferase (AST), alanine aminotransferase (ALT), α-fetoprotein values (AFP), total bilirubin (TBIL) and albumin (ALB). Routine pre-surgical imaging examinations such as abdominal ultrasonography (US), abdominal contrast-enhanced magnetic resonance imaging (MRI) or computed tomography (CT) were also investigated. Additionally, histopathological features including tumor size, number of neoplasms, tumor capsule, satellite lesions, degree of liver cirrhosis (LC), microvascular invasion (MVI) and lymph node metastasis (LNM) were also collected.

### Pre-operative AVT, follow-up and clinical outcomes

In this study, pre-operative AVT was defined as continuous use of at least one type of AVT agent before R0 liver resection including adefovir, entecavir, interferon, lamivudine and telbivudine as reported previously 19. The enrolled patients were consecutively visited every 2 months for two years after the surgery and every 3 to 6 months thereafter. The follow-up examinations were conducted using laboratory tests (serum AFP and liver function) and imaging examination. In addition, we compared the dynamic serum HBV-DNA loading level in pre-operative AVT group and non-AVT group in the PSM match cohort of HBV-HCC patients at four time points including the initial time before AVT, the time before surgery, the time after surgery and the time of last follow-up. Diagnosis of recurrence was based on imaging findings and elevated serum AFP levels. Overall survival (OS) and recurrence-free survival (RFS) were the main outcomes, and the OS was defined as the duration from the date of surgery to the date of death or to the date of last follow-up. RFS was defined as the time between the date of surgery to the date when recurrent HCC was first diagnosed.

### Statistical analysis

Continuous variables, reported as means with standard deviations (SD), were compared using student's *t*-test or Mann-Whitney *U* test. Categorical data, presented as frequencies (%), were compared using Chi-square test or Fisher's exact test. Propensity score (PS) model was constructed to balance the differences of potential confounding variables. And all the variables associated with the AVT group in the univariate analyses with a threshold of *P<*0.1 were covered when constructing the PS model. Then, PSM method was used to reduce the standard mean differences (SMDs) of the covariates. Finally, individuals in the non-AVT group were matched with those in the AVT group using a matching ratio of approximately 1:1 with the closest estimated PS values within 0.1 as reported previously 20. Additionally, Kaplan-Meier curves were adopted to visualize the comparison of OS and RFS between the two groups. Landmark survival analysis was performed to investigate the short or long-term effects of AVT based on the matched data 21. Univariate and multivariate Cox regression analyses were used to determine the independent risk factors of OS and RFS for patients undergoing pre-operative AVT. Stratified analyses were carried out when PSM-matched patients were sub-grouped according to a series of key factors including HBV-DNA loading, HBeAg, esophageal and gastric varices (EGV), AFP, tumor size, LC and MVI to explore the heterogeneity of the potential benefit of pre-operative AVT. R packages including “glm”, “Matching”, “survival” and “tableone” were also used in R (R Foundation for Statistical Computing, Vienna, Austria, and version 3.6.0) and *P<*0.05 indicated significant statistical differences.

## Results

### Patient characteristics

**Figure 1:**
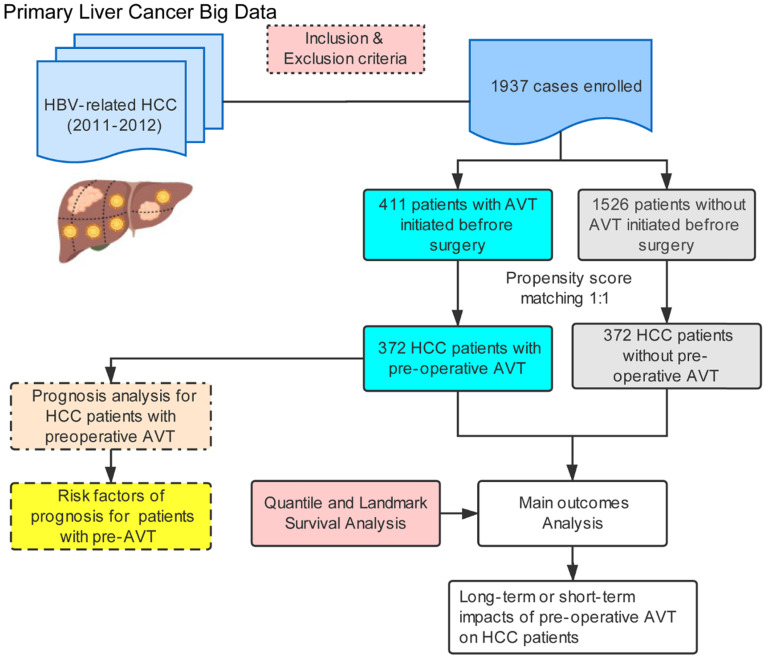
Flow diagram of the study.

The flowchart of this study was illustrated in **Figure 1.** Among 1937 eligible patients, only 411 individuals ever received pre-operative AVT (AVT group) and the other 1526 patients did not (non-AVT group). **Table 1** demonstrates the baseline characteristics of patients from both groups in the unmatched and PSM-matched cohorts. Before matching, there were no significant differences between the two groups in age, sex, history of drinking and smoking, DM, presence of ascites, TBIL, ALB, active hepatitis, LNM, multiple tumors, and tumor capsule. In addition, compared with the non-AVT group, more patients in the AVT group had a family history of HCC, presence of EGV and LC, positive HBeAg, presence of MVI, and BCLC stage of 0 and A. Patients in the AVT group had a lower percentage of high HBV-DNA load (>2000 copies), high AFP level (>400 ng/mL), high AST level (>40 U/L), high ALT level (>40 U/L), presence of satellite lesions, tumor size (>5 cm), B stage of Child-Pugh score. After PSM, a total of matched 744 patients (372 in each group) were further recruited to elucidate the effects of pre-operative AVT on the prognosis after surgery. All variables of the two matched groups were balanced, and *P* values >0.05 indicated the successful matching (**Table 1 and Supplementary Table S1**).

### Treatments and clinical outcomes in PSM-matched cohort

As demonstrated in **Table 2**, the mean AVT duration before diagnosis and surgery was 12.4 weeks, and the initial medications for the pre-operative AVT group in the PSM-matched cohort included lamivudine (n=159, 42.7%), adefovir (n=132, 35.5%), entecavir (n=52, 13.9%), lamivudine plus adefovir (n=20, 5.4%) and telbivudine (n=9, 2.4%). Furthermore, based on the 2000 copy cut-off of HBV-DNA loading, we divided the HBV-HCC patients into two groups (HBV-DNA loading >2000 copies, and ≤ 2000 copies). We found that the positive rate of HBV-DNA in pre-operative AVT group was continuously less than those in the pre-operative non-AVT group at four different time points (**Table 2, Supplementary Figure S1,** *P<*0.05). Both the total death rate (38.4% vs. 30.6%) and the total recurrence rate (59.4% vs. 45.7%) of non-AVT groups were higher than those of the AVT group (**Table 2,** *P<*0.05). In addition, the therapeutic modalities for recurrent HCC were also taken into consideration. The two main strategies were transcatheter arterial chemoembolization (TACE) and surgery, and the others included sorafenib, percutaneous radiofrequency ablation (RFA), radiotherapy, and supportive care. No significant difference was found between the two groups (*P=*0.876).

### Survival and landmark survival analysis

**Figure 2:**
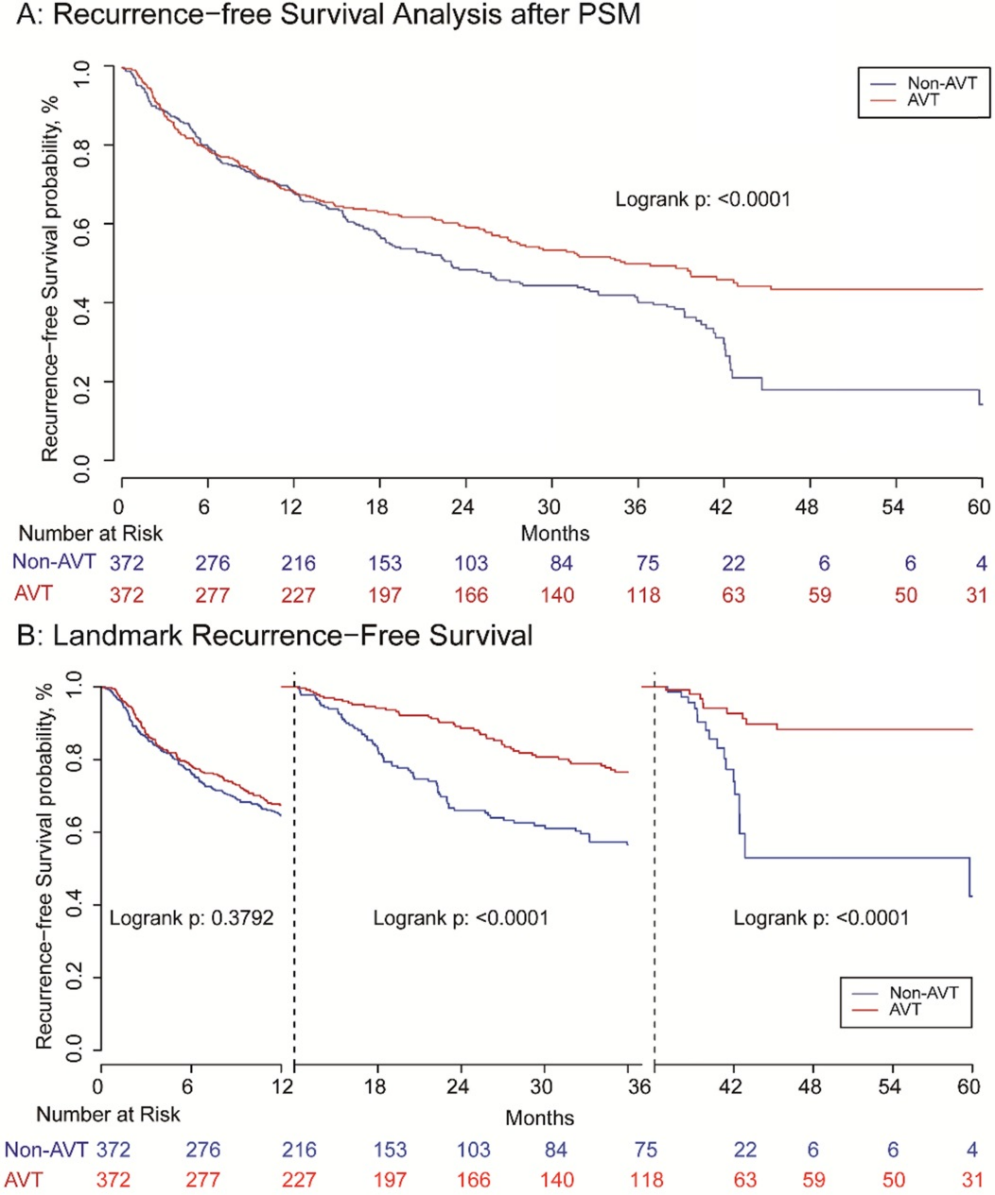
Recurrence-free survival analysis (A) and landmark recurrence-free survival analysis (B) with one and three landmark point.

**Figure 3:**
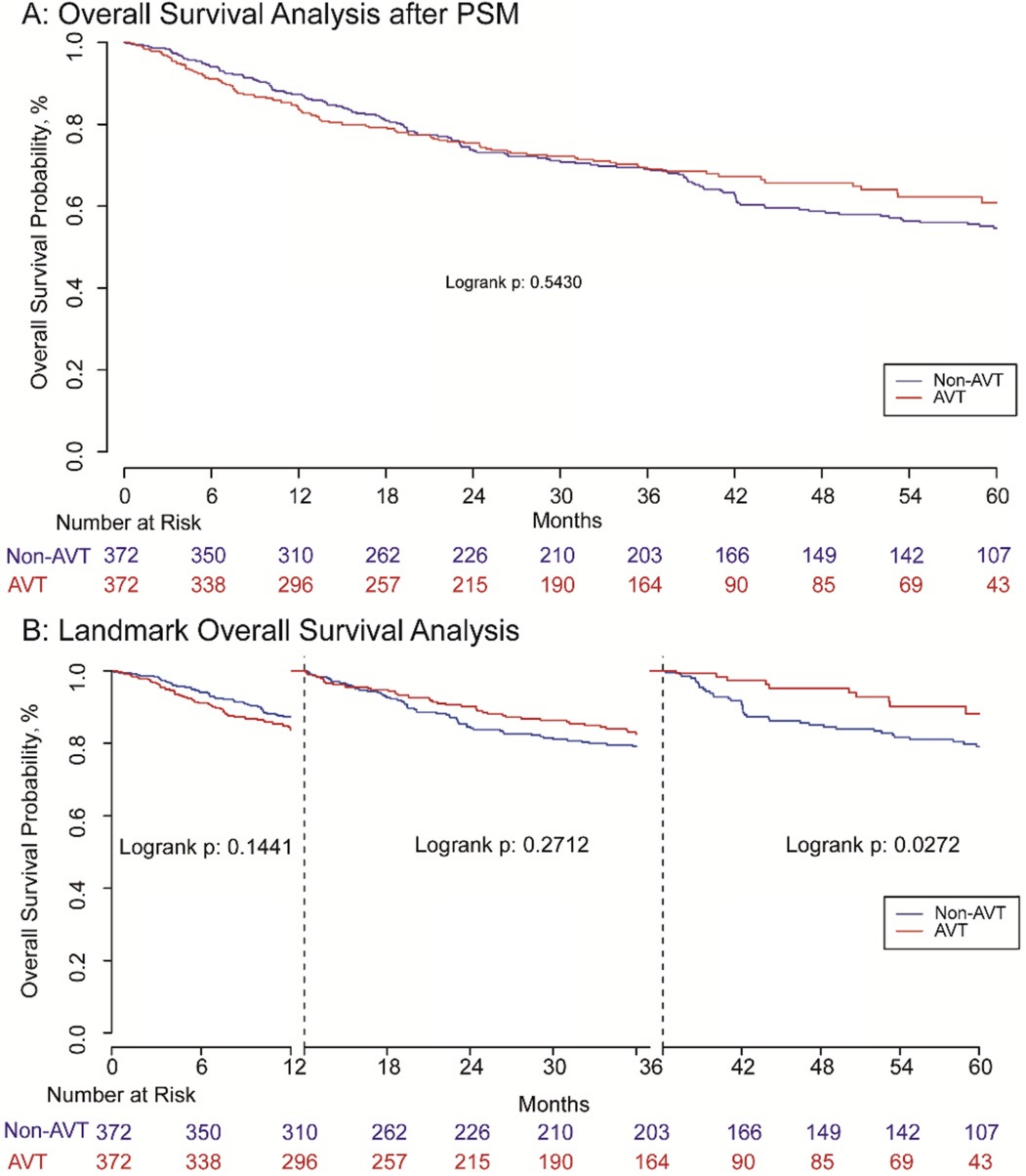
Overall survival analysis (A) and landmark overall survival analysis (B) with one and three landmark point.

Before matching, the RFS of patients receiving pre-operative AVT was better than those in the non-AVT group. The 1, 3, 5-year RFS rate of AVT group was 68.0%, 51.2%, and 43.4%, while the corresponding rate of the non-AVT group was 63.1%, 38.0%, and 18.8%, respectively (*P<*0.001) (**Supplementary Figure S2A**). For OS, the 1, 3, 5-year OS rate of the pre-operative AVT group and the non-AVT group was 83.1%, 70.0%, and 62.4% vs. 82.0%, 62.2% and 48.5%, respectively (*P<*0.001) (**Supplementary Figure S2B**). The mean follow-up time of AVT and non-AVT groups in the matched cohort was 44.15 and 33.78 months, respectively. After matching, patients in the AVT group also presented better RFS than those in the non-AVT group, and the 1, 3, 5-year RFS rates were 67.3%, 49.0%, and 43.1% vs. 66.7%, 41.1% and 18.5% (*P<*0.001) (**Figure 2A**). However, landmark survival analysis in the PSM cohort indicated that in the first one year after surgery, there was no statistical significance of RFS between the two groups (*P=*0.957). After the first 12 months, the AVT group presented better RFS compared with the non-AVT group (*P<*0.001) (**Figure 2B**). As for OS, although the total death rates of the two groups were significantly different (**Table 2,** *P=*0.031), there was no significant difference for OS between the two groups (*P=*0.543) demonstrated by Kaplan-Meier analysis (**Figure 3A**). The landmark survival analysis illustrated that the AVT group did not present better OS than the non-AVT group (*P=*0.027) until 36 months after surgery in the PSM cohort (**Figure 3B**).

### Stratified and subgroup analyses

**Figure 4:**
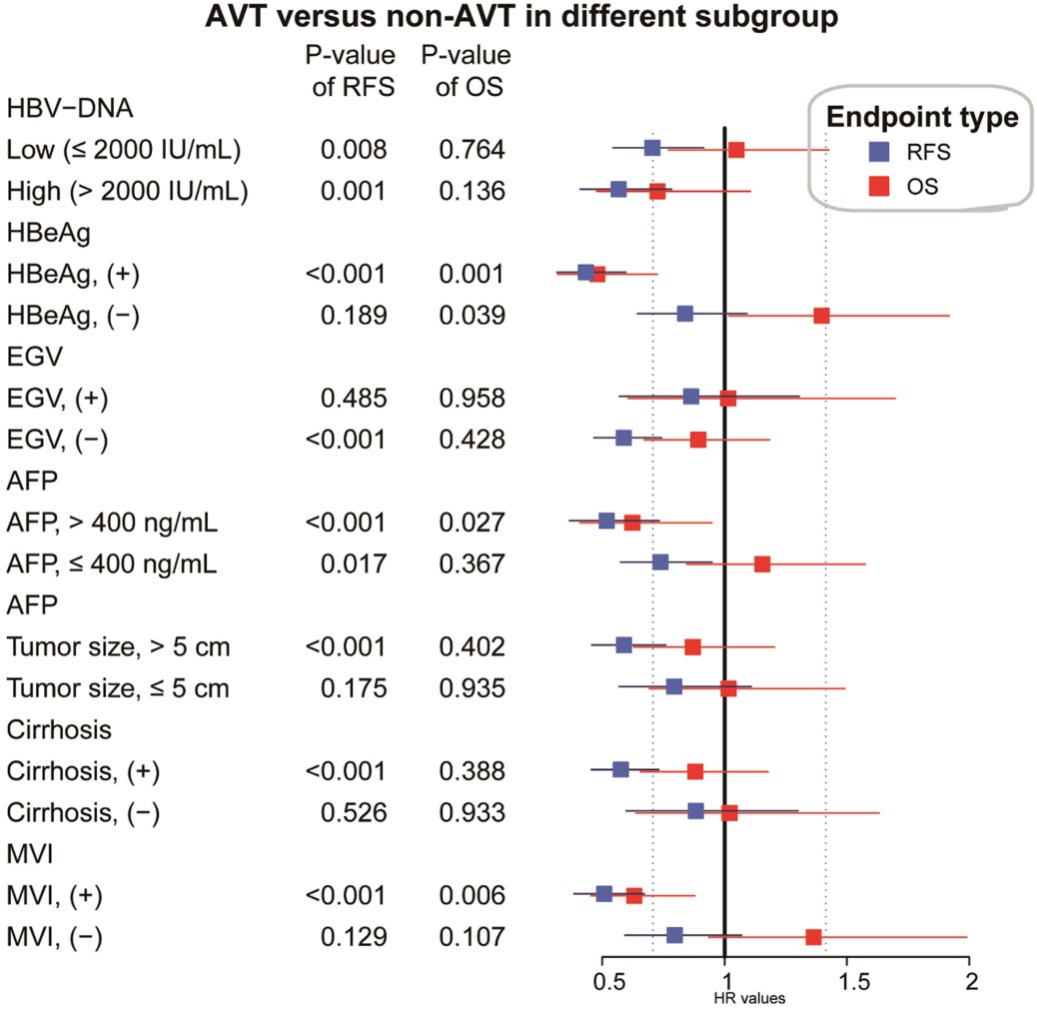
Stratified and subgroup analysis of AVT versus non-AVT in the HCC cohort after PSM (AVT, antiviral treatment, HR, hazard ratio; CI, confidence interval; AFP, α-fetoprotein; MVI, microvascular invasion; EGV, esophageal and gastric varices).

In the PSM matched cohort, the effects of pre-operative AVT on prognosis were further assessed and compared when the participants were stratified by several key clinicopathological factors, including HBV-DNA load, HBeAg, EGV, AFP, tumor size, cirrhosis and MVI. As presented in **Figure 4,** pre-operative AVT was a protective factor of RFS for patients with high or low load of HBV-DNA load, high or low level of AFP, positive HBeAg, tumor size (>5 cm), cirrhosis, MVI, and for those without EGV (all *P<*0.05). In addition, pre-operative AVT could improve OS for patients with positive HBeAg, high level of AFP (>400 ng/mL) and MVI (all *P<*0.05).

### Independent risk factor identification for patients in the AVT group

**Figure 5:**
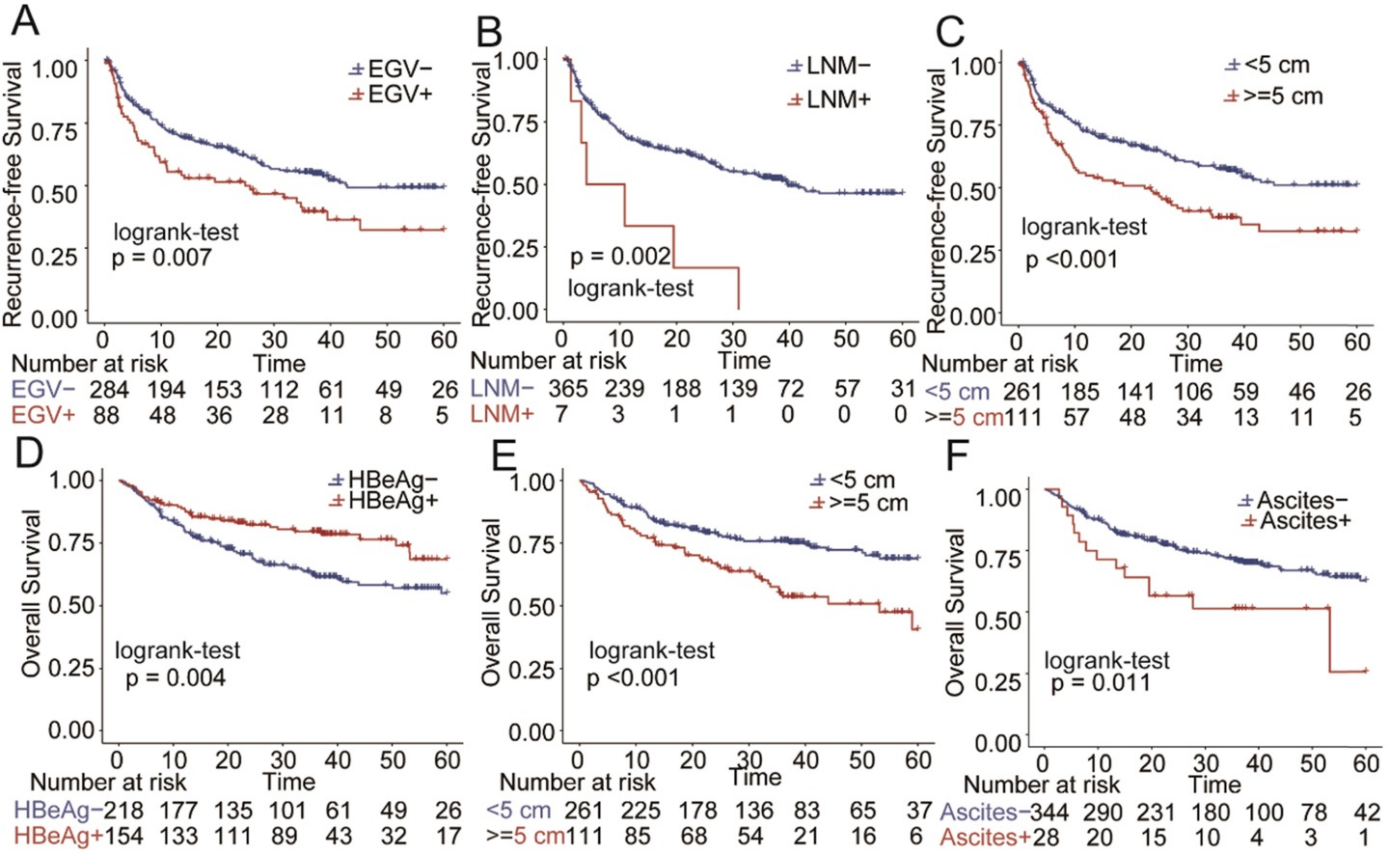
Comparison of overall survival (OS) and recurrence-free survival (RFS) for risk factors in the cohort after PSM. A) esophageal and gastric varices (EGV) in RFS B): lymph node metastasis(LNM) in RFS C):Tumor size in RFS; D) HBeAg in OS; E): Tumor size in OS;F): Ascites in OS.

To identify the independent risk factors affecting RFS and OS of patients who received AVT before surgery, univariate and multivariate Cox regression analyses were performed using the data after PSM. The results of univariate Cox regression analysis are shown in **Supplementary Table S2.** As listed in **Table 3 and Figure 5,** tumor size (>5 cm), EGV and LNM were the independent risk factors of RFS, and tumor size (>5 cm) and ascites were the independent risk factors of OS. In addition, positive HBeAg was an independent protective factor of OS.

## Discussion

It has been widely accepted that therapy for CHB could reduce the risk of HCC, and a relevant predictive score has been developed and validated to help assess risk and make evidence-based decisions accordingly 22, 23. Apart from treatment of the cause of chronic liver disease, no drugs are known to reduce the incidence of HCC, but the existing modalities of AVT still could not eradicate the risk of HBV-induced HCC. Accumulating evidence supported that post-operative AVT could improve the prognosis of HBV-related HCC, and most patients might follow the clinician's advice and take drugs regularly after receiving hepatectomy. However, the effect of AVT on the prognosis for those who received it before surgery has not been elucidated. In addition, conducting longitudinal cohort studies would not be ethical, as no patients were willing to assume the risk of being randomly assigned to the non-AVT group. Here, a retrospective study was conducted to clarify the significance of pre-operative AVT, emphasizing the necessity and significance of regular intaking and good coherence.

In this study, a large cohort (n=1937) consisting of an AVT group (n=1526) and a non-AVT group (n=411) was involved to assess the prognostic impact of pre-operative AVT. Despite the expanding indications, reinforced vaccination and education for AVT recently, most HBV-related HCC patients (nearly 80% in the present research) still haven't received any AVT before diagnosis and initial treatment of PLC. Significant differences of RFS and OS were found between the AVT and non-AVT groups (both *P<*0.01, supplementary data), and benefits of AVT were significant. Furthermore, to minimize the impact of other covariates, PSM was performed under this nonrandomized condition to investigate the effect of AVT on prognosis using matched and balanced data (n=372). Interestingly, after PSM, RFS was significantly different between AVT and non-AVT groups (*P<*0.01), but landmark analysis discriminated between events occurring before and after the first 12 months of follow-up. There's no statistical difference in OS between the two groups after PSM, but the landmark analysis indicated discrimination after 36 months (*P=*0.027). Thus, pre-operative AVT did impact on the late RFS and OS rates.

Furthermore, when participants were stratified by several widely-recognized risk factors including HBV, cirrhotic background, MVI, tumor size and AFP, AVT presented protective effects on RFS in most subgroups and on OS in certain subgroups, particularly, for those patients with MVI and high AFP level. For those with history of AVT before surgery, tumor size, esophageal and gastric varices and LNM were independent risk factors of RFS, while HBeAg, tumor size and ascites were independent risk factors of OS (**Table 3**).

Previous studies have reported that hepatic resection could reactivate HBV replication, especially in patients who did not receive any AVT 24-26. Moreover, pre-operative AVT has been proved to inhibit viral reactivation and contribute to better long-term survival outcomes in patients who underwent repeated hepatectomy for recurrent cases 27. In those cases, AVT could also be described as post-operative. Hence, we assumed that AVT initiated before surgery could also alleviate surgery-induced viral reactivation to some extent.

In addition, compared with non-AVT group, patients in AVT group presented lower levels of aminotransferases, lower incidence of satellite lesions, smaller tumor size and lower BCLC stages (**Table 1**), indicating that AVT might alleviate hepatic inflammatory reaction, relieve the deterioration of liver function, slow down the progression of fibrosis or cirrhosis and improve patients' tolerance to surgical injuries.

In recent decades, a histopathological feature, MVI, has been widely regarded by some guidelines as a poor prognosis indicator once detected in tissue specimens, and prediction of MVI has become a hot topic in HCC research field 28-30. The association of HBV infection or a high level of HBV-DNA with the presence of MVI especially in non-AVT patients have been studied by some groups, and the results in our study indicated the protective effect of AVT on RFS and OS for patients with MVI (**Figure 4**) 31, 32. Nevertheless, whether AVT can prevent the occurrence of MVI or what is the minimum effective medication duration still needs further exploration and validation. Li et al. has found that AVT administered more than 90 days before surgery was related with reduced MVI and early recurrence after partial hepatectomy 19. Interestingly, AVT initiated either before or after surgery has also been emphasized on intrahepatic cholangiocarcinoma (ICC), another important histological type of PLC, as it could reduce recurrence and prolong the OS 33.

As end-stage liver disease, cirrhosis and HCC are common and progressive, and could be considerably ameliorated by AVT, which should be the core for management of CHB. However, the optimal timing of initiating, shifting or stopping AVT for individual patients remains controversial. Recently, a simple score termed TREAT-B based on HBeAg and ALT for selecting HBV treatment candidates in Africa has been developed and deserves further validation 34.

Despite sustained improvements in testing new, efficacious systemic therapies for HCC, AVT still need more initiative and better compliance. For CHB patients, abstaining from therapy before HBsAg loss may lead to less benefits 35. In addition, AVT was safe and efficacious for HCV-related HCC patients awaiting liver transplantation (LT), and could spare organs and benefit patients with a more urgent need 36. In the long run, anti-HBV treatment is also cost-effective.

Relevant mechanisms of pre-operative AVT benefitting short- and long-term prognosis of HBV-related HCC patients were not clear to date, and the optimal pre-operative time window for AVT to affect post-operative outcome remains to be determined. The benefit is a comprehensive result involving heterogeneity and characteristics of tumors, viral and host factors, along with influences of antiviral agents. More emphases should be attached to AVT and compliance for HBV-infected persons as the indication has been gradually expanded over the past years, and more data that can guide clinical practice for precision AVT schemes are necessary.

## Supplementary Material

Supplementary figures and tables.

## Figures and Tables

**Table 1:** Baseline characteristics of hepatocellular carcinoma patients in AVT and non-AVT groups before and after matching

Clinical variables	Before matching				After matching			
	No-AVT (n=1526)	AVT (n=411)	*P* value	SMD	No-AVT (n=372)	AVT (n=372)	*P* value	SMD
Age, years, mean (SD)	50.35 (10.7)	49.95 (9.7)	0.490	0.039	50.42 (11.26)	50.23 (9.67)	0.801	0.018
***Sex, n (%)***			1.000	0.001			1.000	<0.001
male	1299 (85.12%)	350 (85.16%)			318 (85.48%)	318 (85.48%)		
female	227 (14.88%)	61 (14.84%)			54 (14.52%)	54 (14.52%)		
***Drinking, n (%)***			0.297	0.062			0.725	0.032
yes	382 (25.03%)	92 (22.38%)			81 (21.77%)	86 (23.12%)		
no	1144 (74.97%)	319 (77.62%)			291 (78.23%)	286 (76.88%)		
***Family history of HCC, n (%)***			0.016	0.131			0.228	0.100
yes	65 (4.26%)	30 (7.30%)			19(5.11%)	28 (7.53%)		
no	1461 (95.74%)	381 (92.70%)			353(94.89%)	344 (92.47%)		
***DM, n (%)***			0.050	0.109			0.305	0.086
yes	75 (4.91%)	31 (7.54%)			21 (5.65%)	29 (7.80%)		
no	1451(95.09%)	380 (92.46%)			351 (94.35%)	343 (92.20%)		
***Smoking, n (%)***			0.792	0.018			0.137	0.115
yes	518 (33.94%)	143 (34.79%)			111 (29.84%)	131 (35.22%)		
no	1008 (66.06%)	268 (65.21%)			261 (70.16%)	241 (64.78%)		
**Imaging results**								
EGV, n (%)			<0.001	0.220			0.213	0.098
yes	235 (15.40%)	99 (24.09%)			73 (19.62%)	88 (23.66%)		
no	1291 (84.60%)	312 (75.91%)			299 (80.38%)	284 (76.34%)		
***Liver cirrhosis, n (%)***			<0.001	0.319			0.891	0.020
yes	853 (55.90%)	292 (71.05%)			30 (8.06%)	28 (7.53%)		
no	673 (44.10%)	119 (28.95%)			342 (91.94%)	344 (92.47%)		
***Ascites, n (%)***			0.770	0.022			1.000	<0.001
yes	128 (8.39%)	32 (7.79%)			264 (70.97%)	264 (70.97%)		
no	1398 (91.61%)	379 (92.21%)			108 (29.03%)	108 (29.03%)		
**Serological results**								
***HBeAg (+), n (%)***			<0.001	0.227			0.293	0.083
yes	501 (32.83%)	180 (43.80%)			139 (37.37%)	154 (41.40%)		
no	1025 (67.17%)	231 (56.20%)			233 (62.63%)	218 (58.60%)		
***HBV-DNA load, >2000 copies, n (%)***			<0.001	0.758			0.401	0.067
yes	1016 (66.58%)	128 (31.14%)			140 (37.63%)	128 (34.41%)		
no	510 (33.42%)	283 (68.86%)			232 (62.37%)	244 (65.59%)		
***TBIL, n (%)***			0.582	0.034			0.732	0.031
>20 μmol/L	394 (25.82%)	100 (24.33%)			87 (23.39%)	92 (24.73%)		
≤20 μmol/L	1132 (74.18%)	311 (75.67%)			285 (76.61%)	280 (75.27%)		
***AFP, n (%)***			0.002	0.182			0.430	0.064
>400 ng/mL	568 (37.22%)	118 (28.71%)			123 (33.06%)	112 (30.11%)		
≤400 ng/mL	958 (62.78%)	293 (71.29%)			249 (66.94%)	260 (69.89%)		
***ALB, n (%)***			0.376	0.058			0.861	0.026
<30 mg/mL	82 (5.37%)	17 (4.14%)			18 (4.84%)	16 (4.30%)		
≥30 mg/mL	1444 (94.63%)	394 (95.86%)			354 (95.16%)	356 (95.70%)		
***AST, n (%)***			<0.001	0.275			0.826	0.022
>40 U/L	924 (60.55%)	193 (46.96%)			188 (50.54%)	184 (49.46%)		
≤40 U/L	602 (39.45%)	218 (53.04%)			184 (49.46%)	188 (50.54%)		
***ALT, n (%)***			<0.001	0.336			0.329	0.077
>40 U/L	789 (51.70%)	145 (35.28%)			153 (41.13%)	139 (37.37%)		
≤40 U/L	737 (48.30%)	266 (64.72%)			219 (58.87%)	233 (62.63%)		
**Pathological results**								
***Active hepatitis, n (%)***			0.534	0.160			0.572	0.078
yes	826 (54.13%)	185 (45.01%)			153 (41.13%)	158 (42.47%)		
no	700 (45.87%)	226 (54.99%)			219 (58.87%)	214 (57.53%)		
***Satellites lesions, n (%)***			0.001	0.201			0.479	0.059
yes	306 (20.05%)	52 (12.65%)			62 (16.67%)	54 (14.52%)		
no	1220 (79.95%)	359 (87.35%)			310 (83.33%)	318 (85.48%)		
***LNM, n (%)***			0.875	0.020			0.543	0.067
yes	34 (2.23%)	8 (1.95%)			4 (1.08%)	7 (1.88%)		
no	1492 (97.77%)	403 (98.05%)			368 (98.92%)	365 (98.12%)		
***Multiple tumors, n (%)***			0.581	0.036			1.000	<0.001
yes	173 (11.34%)	42 (10.22%)			40 (10.75%)	40 (10.75%)		
no	1353 (88.66%)	369 (89.78%)			332 (89.25%)	332 (89.25%)		
Tumor size, cm, mean (SD)	6.08 (0.40%)	4.52 (1.10%)	<0.001	0.414	4.97 (1.34%)	4.72 (1.27%)	0.324	0.072
***Tumor size, n (%)***			<0.001	0.460			0.579	0.047
>5 cm	752 (49.28%)	113 (27.49%)			119 (31.99%)	111 (29.84%)		
≤5 cm	774 (50.72%)	298 (72.51%)			253 (68.01%)	261 (70.16%)		
***MVI, n (%)***			<0.001	1.177			0.990	0.016
yes	292 (19.13%)	229 (55.72%)			191 (51.34%)	190 (51.08%)		
no	1234 (80.87%)	182 (44.28%)			181 (48.66%)	182 (48.92%)		
***Tumor capsule, n (%)***			0.145	0.131			0.951	0.043
no	447 (29.29%)	99 (24.09%)			97 (26.08%)	91 (24.46%)		
not complete	550 (36.04%)	158 (38.44%)			145 (38.98%)	145 (38.98%)		
complete	424 (27.79%)	129 (31.39%)			112 (30.11%)	118 (31.72%)		
unknown	105 (6.88%)	25 (6.08%)			18 (4.84%)	18 (4.84%)		
***Child-Pugh stage, n (%)***			0.025	0.148			1.000	0.021
A	1462 (95.81%)	404 (98.30%)			366(98.39%)	365 (98.12%)		
B	64 (4.19%)	7 (1.70%)			6(1.61%)	7 (1.88%)		
***BCLC stage, n (%)***			<0.001	0.412			0.532	0.036
0 stage	85 (5.6)	35 (8.5)			25 (6.7)	30 (8.1)		
A stage	543 (35.6)	215 (52.3)			186 (50.0)	186 (50.0)		
B stage	713 (46.7)	119 (29.0)			119 (32.0)	117 (31.5)		
C stage	185 (12.1)	42 (10.2)			42 (11.2)	39 (10.5)		

**Abbreviations:** AVT, antiviral treatment; DM, diabetes mellitus; SMD, standard mean differences; AFP, α-fetoprotein; ALB, albumin, AST, aspartate aminotransferase; ALT, alanine aminotransferase; EGV, esophageal and gastric varices; TBIL, total bilirubin, LNM, lymph node metastasis; MVI, microvascular invasion; BCLC, Barcelona Clinic Liver Cancer.

**Table 2:** Treatments and outcomes in PSM matched cohort

Variables	Non-AVT	AVT	*P* value	SMD
	n=372	n=372		
AVT duration, week, mean (SD)	NA	12.4 (8)	NA	NA
**Antiviral Drugs, n (%)**			NA	NA
Adefovir	NA	132 (35.5)		
Entecavir	NA	52 (14.0)		
Lamivudine	NA	159 (42.7)		
Lamivudine + Adefovir	NA	20 (5.4)		
Telbivudine	NA	9 (2.4)		
**Dynamic HBV-DNA levels, <2000 copies, n %**				
Not started of AVT	195 (52.4)	189 (50.8)	0.660	0.018
Before Surgery	/	241 (64.8)	0.001	0.221
After Surgery	189 (50.8)	229 (61.6)	0.003	0.205
The last test of follow-up	290 (78.0)	312 (83.9)	0.040	0.145
Total death rate, n (%)	143 (38.4)	114 (30.6)	0.031	0.164
0-1 year	47 (12.6)	60 (16.1)		
1-2 year	44 (11.8)	27 (7.3)		
2-3 year	14 (3.8)	12 (3.2)		
3-5year	38 (10.2)	10 (2.7)		
Total recurrence rate, n (%)	221 (59.4)	170 (45.7)	<0.001	0.277
0-1 year	128 (34.4)	116 (31.2)		
1-2 year	63 (16.9)	24 (6.5)		
2-3 year	13 (3.5)	21 (5.6)		
3-5year	17 (4.6)	9 (2.4)		
**Treatment for recurrent HCC, n (%)**			0.876	0.023
Surgical resection	33 (8.9)	26 (7.0)		
TACE	123 (33.1)	95 (25.5)		
Sorafenib	13 (3.5)	12 (3.2)		
RFA	15 (4.0)	10 (2.7)		
Radiotherapy	1 (0.3)	5 (1.3)		
Supportive care	36 (9.7)	22 (5.9)		

**Abbreviations:** PSM, propensity score matching; OS, overall survival; RFS, recurrence-free survival; AVT, antiviral treatment; TACE, transcatheter arterial chemoembolization; RFA, percutaneous radiofrequency ablation. SMD, standard mean differences.

**Table 3:** Multivariate cox regression analyses of AVT group data after PSM

	HR	95%CI	*P* value
**RFS**			
Tumor size, >5 vs. ≤ 5 cm	1.666	1.212-2.290	0.002
EGV, positive vs. negative	1.494	1.069-2.087	0.019
LNM, positive vs. negative	4.049	1.773-9.246	0.001
**OS**			
HBeAg, positive vs. negative	0.525	0.346-0.797	0.002
Tumor size, >5 vs. ≤ 5 cm	1.891	1.292-2.769	0.001
Ascites, yes vs. no	2.117	1.219-3.677	0.008

**Abbreviations:** RFS, recurrence-free survival; LNM, lymph node metastasis; OS, overall survival; EGV, esophageal and gastric varices.
