# Supplementary material for: Distinct Impacts of Pre-Operative Antiviral Treatment on Post-Operative Outcomes of HBV-related Hepatocellular Carcinoma: A Landmark Analysis
Source: J Cancer. 2021 Jan 1;12(1):170–80. doi: 10.7150/jca.47125 (PMC7738838; doi:10.7150/jca.47125)
Supplement: Supplementary file 1 — Supplementary figures and tables. [file jcav12p0170s1.pdf]

Supplementary materials

Supplementary Table S1. Propensity score modeling for AVT patients

Variables	HR	95% CI	P value
Age, >60 vs. ≤60 years	0.992	0.980-1.004	0.213
Sex, male vs. female	1.097	0.767-1.571	0.610
Esophageal and gastric varices, yes vs. no	1.514	1.107-2.072	0.010
AFP >400 ng/mL, yes vs. no	0.711	0.536-0.943	0.018
Albumin <30 mg/ml, yes vs. no	1.206	0.617-2.357	0.584
AST >40 U/L, yes vs. no	0.900	0.686-1.182	0.450
ALT >40 U/L, yes vs. no	0.701	0.531-0.926	0.012
HBsAg (+), yes vs. no	2.878	0.803-10.308	0.104
HBeAg (+), yes vs. no	2.442	1.856-3.214	<0.001
HBV DNA, >2000 vs. ≤2000 copies	0.202	0.153-0.266	<0.001
Tumor size, >5 vs. ≤5 cm	0.568	0.383-0.842	0.005
Child-Pugh, B vs. A	0.355	0.134-0.941	0.037
Multiple tumors, yes vs. no	0.159	0.075-0.340	<0.001
Total bilirubin >20, μmol/l, yes vs. no	0.979	0.731-1.311	0.885
Cirrhosis, yes vs. no	0.188	0.130-0.271	<0.001
Ascites, yes vs. no	0.737	0.508-1.070	0.108
MVI, yes vs. no	0.977	0.740-1.291	0.872
Satellites, yes vs. no	1.158	0.719-1.865	0.546
Tumor capsule, yes vs. no	1.203	1.043-1.388	0.012
BCLC stage			
A vs. 0	1.586	0.514-4.894	0.423
B vs. 0	1.155	0.354-3.773	0.811
C vs. 0	1.069	0.319-3.583	0.914

Abbreviations: AVT, antiviral treatment; AFP, α-fetoprotein; AST, aspartate aminotransferase; ALT, alanine aminotransferase; LNM, lymph node metastasis; MVI, microvascular invasion; BCLC, Barcelona Clinic Liver Cancer.

Supplementary Figure S1. Comparison of dynamic HBV-DNA loading in AVT group and none-AVT group after PSM

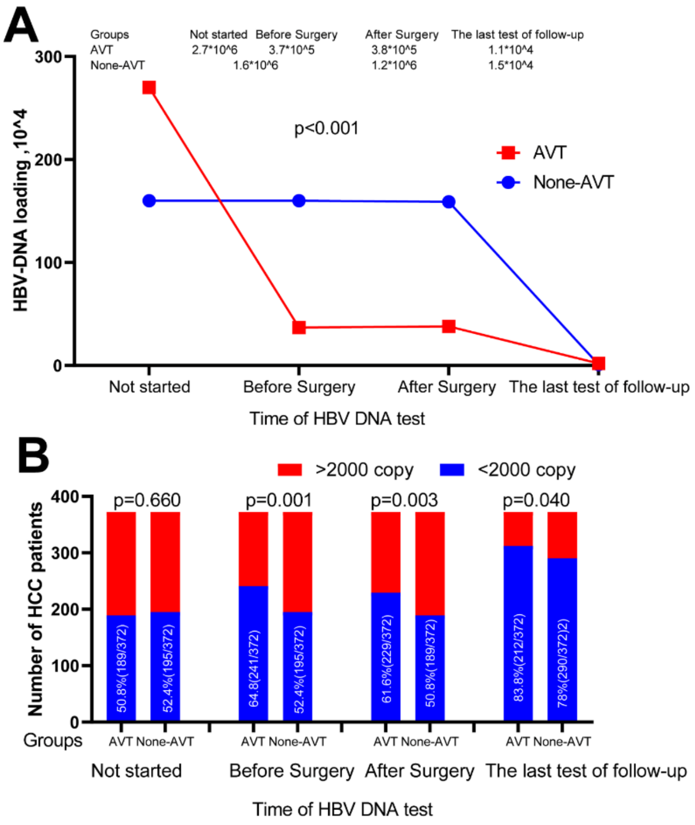

Supplementary Figure S2. Survival analysis before PSM

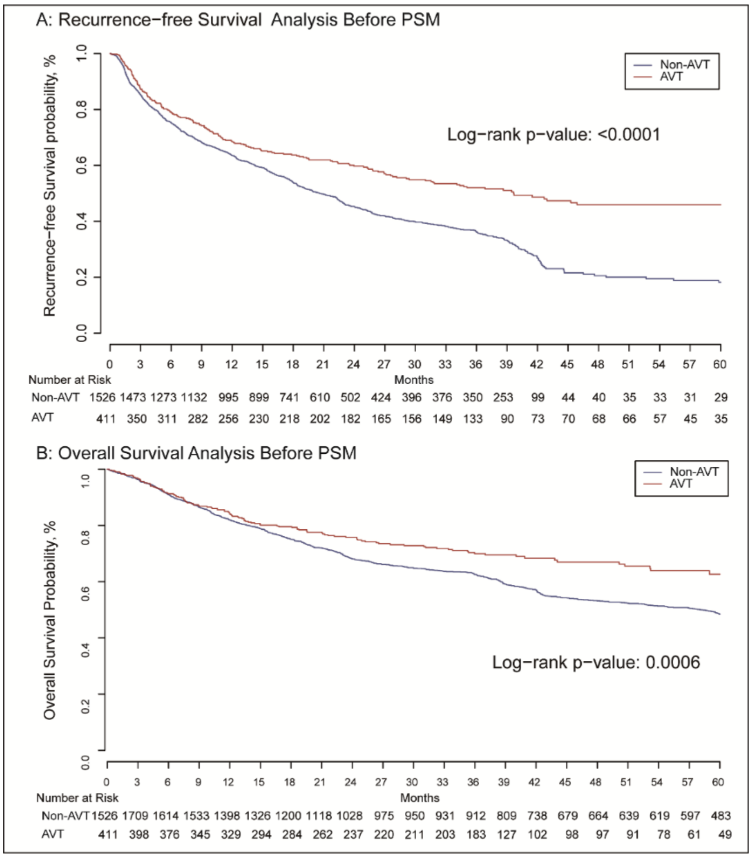

Supplementary Table S2. Univariate cox regression analysis for RFS and OS in the AVT group after PSM (n=372)

Variables	RFS			OS		
	HR	95%CI	P value	HR	95%CI	P value
Age, >60 years vs. ≤60 years	1.013	0.997-1.029	0.117	1.010	0.990-1.029	0.324
Sex, male vs. female	1.623	0.954-2.760	0.074	0.953	0.553-1.644	0.863
Child-Pugh, B vs. A	1.000	1.000-1.000	0.462	1.253	0.856-1.835	0.247
Drinking, yes vs. no	0.852	0.584-1.242	0.404	1.091	0.707-1.684	0.694
Family History of HCC, yes vs. no	1.449	0.888-2.365	0.137	0.772	0.359-1.659	0.507
Diabetes mellitus, yes vs. no	0.877	0.487-1.579	0.663	1.591	0.908-2.789	0.105
Smoking, yes vs. no	1.168	0.851-1.603	0.335	1.094	0.742-1.614	0.650
AFP >400 ng/mL, yes vs. no	1.074	0.769-1.500	0.674	1.05	0.698-1.580	0.814
Albumin <30 mg/ml, yes vs. no	0.819	0.384-1.746	0.605	0.347	0.086-1.407	0.138
AST >40 U/L, yes vs. no	1.384	1.023-1.872	0.035	1.376	0.945-2.004	0.095
ALT >40 U/L, yes vs. no	0.855	0.621-1.176	0.335	0.826	0.556-1.226	0.342
Total bilirubin >20, μmol/L, yes vs. no	1.364	0.973-1.914	0.072	1.183	0.774-1.809	0.437
HBsAg (+), yes vs. no	1.000	NA	NA	1.000	NA	NA
HBeAg (+), yes vs. no	0.846	0.618-1.158	0.297	0.526	0.347-0.797	0.002
Anti-HCV (+), yes vs. no	0.493	0.122-1.987	0.320	0.756	0.187-3.063	0.695
HBV DNA, >2000 vs. ≤2000 copies	1.067	0.780-1.460	0.683	0.795	0.534-1.184	0.259
Tumor size, >5 cm vs. ≤5 cm	1.727	1.261-2.366	0.001	2.039	1.399-2.971	<0.001
Multiple tumors, yes vs. no	1.198	0.743-1.933	0.459	1.352	0.772-2.370	0.292
Esophageal and gastric varices, yes vs. no	1.794	1.276-2.523	0.001	1.542	1.008-2.359	0.046
Cirrhosis, yes vs. no	1.249	0.882-1.769	0.210	0.959	0.638-1.442	0.842
Ascites, yes vs. no	1.435	0.857-2.404	0.170	2.288	1.326-3.950	0.003
Satellites, yes vs. no	1.292	0.949-1.759	0.103	0.898	0.512-1.575	0.708
MVI, yes vs. no	1.267	0.839-1.915	0.261	1.202	0.824-1.752	0.340
LNM, yes vs. no	3.316	1.464-7.512	0.004	3.591	1.314-9.808	0.013
Tumor capsule, yes vs. no	1.033	0.857-1.247	0.732	1.144	0.913-1.432	0.243

Abbreviations: AVT, antiviral treatment; AFP, α-fetoprotein; AST, aspartate aminotransferase; ALT, alanine aminotransferase; LNM, lymph node metastasis; MVI, microvascular invasion; BCLC, Barcelona Clinic Liver Cancer.
